# A drug‐repositioning screen for primary pancreatic ductal adenocarcinoma cells identifies 6‐thioguanine as an effective therapeutic agent for TPMT‐low cancer cells

**DOI:** 10.1002/1878-0261.12364

**Published:** 2018-08-29

**Authors:** Inki Kim, Yeon‐Sook Choi, Jae Hwi Song, Eun A Choi, Sojung Park, Eun Ji Lee, Je‐Keun Rhee, Song Cheol Kim, Suhwan Chang

**Affiliations:** ^1^ Convergence Medicine Research Center (CREDIT)/Biomedical Research Center Asan Institute for Life Sciences Seoul Korea; ^2^ Department of Convergence Medicine Asan Medical Center University of Ulsan College of Medicine Seoul Korea; ^3^ Department of Biomedical Sciences Asan Medical Center University of Ulsan College of Medicine Seoul Korea; ^4^ Cancer Research Institute Catholic University of Korea Seoul Korea; ^5^ Department of Surgery Asan Medical Center University of Ulsan College of Medicine Seoul Korea; ^6^ Department of Physiology Asan Medical Center University of Ulsan College of Medicine Seoul Korea

**Keywords:** 6‐thioguanine, drug repositioning, pancreatic ductal adenocarcinoma, patient‐derived xenograft model, thiopurine methyltransferase

## Abstract

Pancreatic cancer is one of the most difficult cancers to cure due to the lack of early diagnostic tools and effective therapeutic agents. In this study, we aimed to isolate new bioactive compounds that effectively kill pancreatic ductal adenocarcinoma (PDAC) cells, but not untransformed, human pancreatic ductal epithelial (HPDE) cells. To this end, we established four primary PDAC cell lines and screened 4141 compounds from four bioactive‐compound libraries. Initial screening yielded 113 primary hit compounds that caused over a 50% viability reduction in all tested PDAC cells. Subsequent triplicate, dose‐dependent analysis revealed three compounds with a tumor cell‐specific cytotoxic effect. We found that these three compounds fall into a single category of thiopurine biogenesis. Among them, 6‐thioguanine (6‐TG) showed an IC_50_ of 0.39–1.13 μm toward PDAC cells but had no effect on HPDE cells. We propose that this cancer selectivity is due to differences in thiopurine methyltransferase (TPMT) expression between normal and cancer cells. This enzyme is responsible for methylation of thiopurine, which reduces its cytotoxicity. We found that *TPMT* levels were lower in all four PDAC cell lines than in HPDE or Panc1 cells, and that knockdown of *TPMT* in HPDE or Panc1 cells sensitized them to 6‐TG. Lastly, we used a patient‐derived xenograft model to confirm that 6‐TG has a significant antitumor effect in combination with gemcitabine. Overall, our study presents 6‐TG as a strong candidate for use as a therapeutic agent against PDAC with low levels of TPMT.

**Abbreviations**
6‐TG: 6‐thioguanine
ALL: acute lymphocytic leukemia
AML: acute myeloid leukemia
CML: chronic myeloid leukemia
HPDE: human pancreatic ductal epithelial
MTAP: methylthioadenosine phosphorylase
PDAC: pancreatic ductal adenocarcinoma
PDX: patient‐derived xenograft
TPMT: thiopurine methyltransferase

## 1 Introduction

Despite the recent advances in surgical techniques and other treatment strategies, pancreatic cancer remains a cancer type with the lowest 5‐year survival rate (He and Yuan, 2014; Kim, 2008). The reasons for such poor prognosis include late diagnosis, which makes only 20–25% of the cases operable (Jutric and Melstrom, 2017), as well as fewer options for chemotherapy owing to the refractoriness of pancreatic ductal adenocarcinoma (PDAC) cells toward anticancer drugs (Rajabpour *et al*., 2017). Moreover, there is a desmoplastic reaction, which means accumulation of the extracellular matrix along with activated (fibrotic) stellate cells (Bahrami *et al*., 2017), resulting in a barrier to drug delivery (Lunardi *et al*., 2014). Despite these challenges, there are newly developed therapeutic options, including FOLFIRINOX and Paclitaxel‐Nab, that have shown progress in chemotherapy (Sohal *et al*., 2016). Yet, there is a desperate need for an effective therapeutic agent to extend the survival of more patients with PDAC.

Drug‐repositioning screening has been increasingly performed to identify therapeutic agents for complex diseases (Li and Jones, 2012) because of the high cost and long time required for the development of novel therapeutic agents. For pancreatic cancer, ritonavir, an HIV inhibitor, was identified as an antitumor agent inhibiting cell cycle progression (Batchu *et al*., 2014). Recently, bazedoxifene, which is approved as an estrogen modulator, was revealed as an anti‐GP130 signaling agent that can inhibit pancreatic cancer cell growth (Wu *et al*., 2016). Although these findings reveal novel therapeutic agents based on the cytotoxicity to PDAC cells, often these drugs exert toxicity toward normal cells. Moreover, these novel candidates have usually been screened on human PDAC cell lines such as BxPC‐3, MIA PaCa‐2, and PANC‐1. Considering the heterogeneity of PDAC (Iovanna and Dusetti, 2017), there is a need to apply drug repositioning to other PDAC cell lines including primary cells.

Here, we describe a drug‐repositioning screening involving four primary PDAC cell lines and human pancreatic ductal epithelial (HPDE) cells as a normal cell control. We aimed to find a candidate that exerts superior cytotoxicity toward cancer cells than toward healthy cells. As a result, we suggest thiopurine analogs as such candidates. 6‐TG seems to be a PDAC‐specific antitumor agent, and we confirmed its efficacy in a patient‐derived xenograft model.

## 2 Materials and methods

### 2.1 Cell culture and transfection

Human pancreatic cancer cell lines (Panc1, Mia‐Paca2, and BxPC3) were obtained from ATCC. They were maintained in DMEM containing 10% of FBS and 1% of a penicillin/streptomycin solution (Hyclone, Logan, UT, USA). Pancreatic cancer primary cells were cultured in RPMI with 5% FBS, 1% of the penicillin/streptomycin solution, 20 ng·mL^−1^ EGF, 4 μg·mL^−1^ hydrocortisone, and 4 μg·mL^−1^ transferrin. HPDE cells were cultured in a keratinocyte serum‐free medium supplemented with EGF and bovine pituitary extract (Invitrogen, Thermo Fisher Scientific, Mississauga, ON, Canada). All the cells were cultured at 37 °C and 5% CO_2_ in a humidified incubator. All cell lines were authenticated using short tandem repeat (STR) analysis as described in 2012 in ANSI Standard (ASN‐0002) by the ATCC Standards Development Organization. For transient expression of thiopurine methyltransferase (TPMT) or methylthioadenosine phosphorylase (MTAP), cells were transfected via Lipofectamine^®^ 2000 (Invitrogen) for 3 h, and the cells then assayed 48 h after the transfection.

### 2.2 Derivation of primary culture

Primary cancer cells were isolated from a tumor of a patient and were used to set up a xenograft model. The experiments were undertaken with the understanding and written consent of each subject. The study methodologies conformed to the standards set by the Declaration of Helsinki and were approved by the ethics committee in Asan Medical Center, Seoul, South Korea.

Fresh tumor tissue was minced into 1‐ to 2‐mm pieces using sterile scissors, a scalpel, and forceps. For tissue digestion, the tissue pieces were placed in a 15‐mL conical tube with 3–5 mL of RPMI 1640 medium (PAN Biotech, Aidenbach, Bavaria, Germany) containing 5% FBS (PAN Biotech), 1% of the penicillin/streptomycin solution (Hyclone), 20 μg·mL^−1^ collagenase Type III (Sigma‐Aldrich, Oakville, ON, Canada), and 840 ng·mL^−1^ Fungizone™ (Gibco, Thermo Fisher Scientific). Tissues were kept for 2 h in a 37 °C shaking incubator. After incubation, the digested tissue pieces were washed with the RPMI 1640 medium and centrifuged at 800 r.p.m. for 3 min, three times. The tissue was placed in a T25 flask coated with collagen and cultured in the RPMI 1640 medium containing 5% FBS, 1% of the penicillin/streptomycin solution, 20 ng·mL^−1^ hEGF (Gibco), 4 μg·mL^−1^ hydrocortisone (Sigma‐Aldrich), 4 μg·mL^−1^ transferrin (Sigma‐Aldrich), and 840 ng·mL^−1^ Fungizone™ at 37 °C with 5% CO_2_. After 2–4 days, cells started to become attached to the T25 flask.

### 2.3 Protein extraction and Western blotting

Cells were harvested in RIPA buffer containing protease and phosphatase inhibitors. The lysates were centrifuged at 21 000 ***g*** for 15 min, and the supernatant was collected. Proteins were separated by SDS polyacrylamide gel electrophoresis. Immunoblotting was performed with antibodies to MTAP (Cell Signaling Technology, Danvers, MA, USA), TPMT (Invitrogen) and β‐Actin (Santa Cruz Biotechnology, Dallas, TX, USA), p‐BRAF (Cell Signaling Technology), p‐MEK (Cell Signaling Technology), p‐ERK (Cell Signaling Technology), Caspase‐7 (Cell Signaling Technology), and PARP (Cell Signaling Technology).

### 2.4 RNA preparation and real‐time PCR

RNA extraction was performed by means of TRIzol (Invitrogen). RNA (1 μg) was subjected to cDNA synthesis (PrimeScript RT reagent kit, Takara Bio, Shiga, Japan). Real‐time PCR was performed with SYBR Green (Enzo Life Sciences, Farmingdale, NY, USA), a Bio‐Rad real‐time PCR detection system.

The primers for qRT‐PCR were as follows: *MTAP*, 5′‐AGGGACCTCGTTTTAGCTCC‐3′ and 5′‐GAAACTGCTTCCTCGTGCTC‐3′; *TPMT*, 5′‐ACGGCAAGACTGCTTTTCAT‐3′ and 5′‐CACTGATTTCCACACCAACTACA‐3′; *GAPDH*, 5′‐ACCCAGAAGACTGTGGATGG‐3′ and 5′‐TCTAGACGGCAGGTCAGGTC‐3′.

### 2.5 Plasmids and siRNA treatment

Complete coding sequence of human MTAP and TPMT was inserted into pFlag‐CMV. Sequences of human MTAP and TPMT were amplified from cDNAs by KOD plus polymerase (TOYOBO, Osaka, Japan).

Cells were transfected with negative control, *MTAP* siRNA, and *TPMT* siRNAs via Lipofectamine^®^ 2000 (Invitrogen). Human siRNA was designed by Genolution Inc. (Seoul, Korea) using the following sequences: *MTAP*, 5′‐GUCAACUACCAGGCGAACAUCUU‐3′; *TPMT‐1*, GCGGUUGAGAUGAAAUGGUUUUU; *TPMT‐2*, CUGUGUGUUCUUUCUUAUGAUUU.

### 2.6 1st drug screening

Cells were recovered from cultures using trypsin and re‐plated in 96‐well plates at 4 × 10^3^ cells per well in 195 μL of culture medium, and the plates were returned to the humidified atmosphere and incubated there overnight. Using an automated liquid handler (Janus; Perkin Elmer, Waltham, MA, USA), 5 μL of test library compounds in 10% DMSO was added to wells to achieve a final concentration of 5 μm. After 48 h of incubation, cell viability was assessed by an intracellular ATP content assay (CellTiter Glo, Promega, Fitchburg, WI, USA). The plates were read on a Victor3 (Perkin Elmer) label reader in luminescent mode. Raw values from each plate were transferred and analyzed in prism software (GraphPad Software, La Jolla, CA, USA) to evaluate survival rates.

### 2.7 2nd confirmation screening

The primary hit compounds selected from primary screen were retested at 1‐ and 5‐μm concentrations. Every step used for evaluation of survival rates was performed as per the primary screening protocol.

### 2.8 Determination of IC_50_ values

Cell viability was evaluated using Ezcytox. The data were defined as (mean 6‐TG‐treated A_450_ − blank)/(mean untreated control A_450 _− blank) × 100 and analyzed. The IC_50_ values were determined with the prism software.

### 2.9 *In vivo* drug efficacy test

The animal experiments were performed in accordance with the Korean Ministry of Food and Drug Safety (KMFDS) guidelines. Protocols for animal experiments were reviewed and approved by the Institutional Animal Care and Use Committees (IACUC) of Asan Institute for Life Sciences (Project Number: 2016‐12‐051). All mice were maintained in the specific pathogen‐free (SPF) facility of the Laboratory of Animal Research in the Asan Medical Center. To prepare a patient‐derived xenograft model, all the animals were anesthetized with 15 mg·kg^−1^ Zoletil^®^ (Virbac, Fort Worth, TX, USA) and 2.5 mg·kg^−1^ Rompun^®^ (Bayer Korea Ltd, Seoul, South Korea) i.p. Tumor tissue was sliced into one to two 2‐mm^3^ fragments and implanted into mice subcutaneously. When the tumor volume reached approximately 100 mm^3^, drugs were administered i.p. twice a week (6‐TG, 25 mg·kg^−1^; gemcitabine, 100 mg·kg^−1^). Length (*L*) and width (*W*) of a tumor were measured using calipers, and tumor size was calculated as follows:

$$\text{Tumor}\;\text{size}\;\left({\text{mm}}^{3}\right)=\frac{L\times W^{2}}{2}.$$

### 2.10 Annexin V/propidium iodide staining and FACS analysis

Pancreatic cancer primary cells were seeded in six‐well plates at 50–60% confluence and harvested following treatment with 6‐TG (1 μm) for 48 h. After 48 h, cells were harvested and stained according to the protocol of the FITC‐Annexin V Apoptosis Detection kit (BD Biosciences, Franklin Lakes, NJ, USA). The percentage of apoptotic cell population was detected by Accuri Flow Cytometry (BD Biosciences) and calculated using the cflow software (National Institutes of Health, Bethesda, MD, USA).

### 2.11 Species‐specific PCR

The genomic DNA (gDNA) was extracted from the tumor tissue using the phenol‐chloroform method. DNA concentrations were measured on a NanoDrop. Expression level was analyzed by PCR using human‐ and mouse‐specific primer pairs (Alcoser *et al*., 2011). PCR products were separated by agarose gel electrophoresis, and relative expression was quantified using imagej (National Institutes of Health, Bethesda, MD, USA).

### 2.12 TCGA data analysis

Public RNA‐seq data for informatic analysis were downloaded from Broad GDAC Firehouse (https://gdac.broadinstitute.org). Average TPMT level for each type of tumor was calculated using RSEM value and visualized as a bar chart format. For PDAC, the distribution of TPMT expression is obtained from 180 samples and displayed as a dot graph, by ascending order.

### 2.13 Statistics

Data were analyzed by two‐way anova or Student's *t*‐test and expressed as means ± standard deviation (SD).

## 3 Results

### 3.1 Derivation and characterization of primary PDAC culture from a fresh tumor specimen

**Figure 1:**
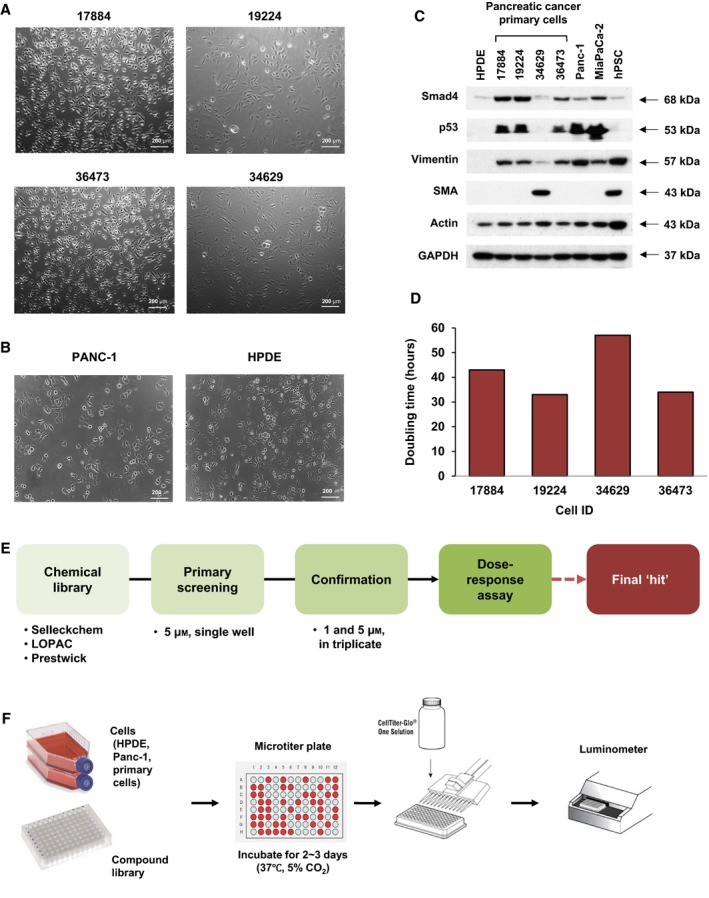
Characteristics of primary PDAC cells and overall workflow of the repositioning screening. (A) Representative pictures of four primary PDAC cells used in the screening. Scale bar: 200 μm. (B) Pictures of Panc1 and HPDE cell lines that served as control cells. (C) Western blot results of Smad4, p53, vimentin, and smooth muscle actin (SMA), which were used as markers of PDAC or stellate cells. Human pancreatic stellate cells (hPSC). Actin and GAPDH served as a loading control. (D) A graph of doubling time for the four primary PDAC cells. (E) A schematic diagram showing steps of drug repositioning. The 1st screening was done at 5 mm as a single point. The subsequent confirmation step was performed at 1 and 5 mm, in triplicate. The last step is to measure IC_50_ of each drug, with an 8‐point serial dilution. (F) An overall scheme of the screening. PDAC and HPDE cells were seeded in a 96‐well plate, and a compound library was added and incubated for 48–72 h. After that, the cell viability was measured with CellTiter‐Glo in a luminometer.

To establish a primary culture for drug screening, we obtained a freshly dissected tumor (from Asan Medical Center, IRB No. S2013‐0744‐0009). Of 21 specimens tested, we were able to culture four primary cell lines. The pictures of primary cells are shown in Fig. 1A. As a normal cell control, we used HPDE cells (Fig. 1B). In contrast, Panc1, which is reported to be a drug‐resistant cell line (Anderson *et al*., 2002; Nguyen *et al*., 2003), was added to the screening (Fig. 1B). The genomic characteristics of the tumor specimen were described previously (Jung *et al*., 2016), and other clinical features are summarized in Table S1. Of the four primary cell clones, the 34 629 cell line showed distinct cellular morphology and molecular features, as depicted in Fig. 1C. Western blot analysis of SMAD4 and P53 (which is frequently mutated in PDAC), as well as vimentin and SMA (myoepithelial cell marker; Mace *et al*., 2013), revealed that the 34 629‐cell line has a stellate cell‐like molecular phenotype but is not exactly the same (as shown in vimentin, Fig. 1C). This finding was confirmed by RT‐PCR analysis of matrix gene expression (Fig. S1). The doubling times of four primary cells selected for drug screening were comparable, measured from its growth curve (Fig. 1D).

### 3.2 Primary screening identified 113 compounds of four drug libraries, effective against PDAC cells

**Figure 2:**
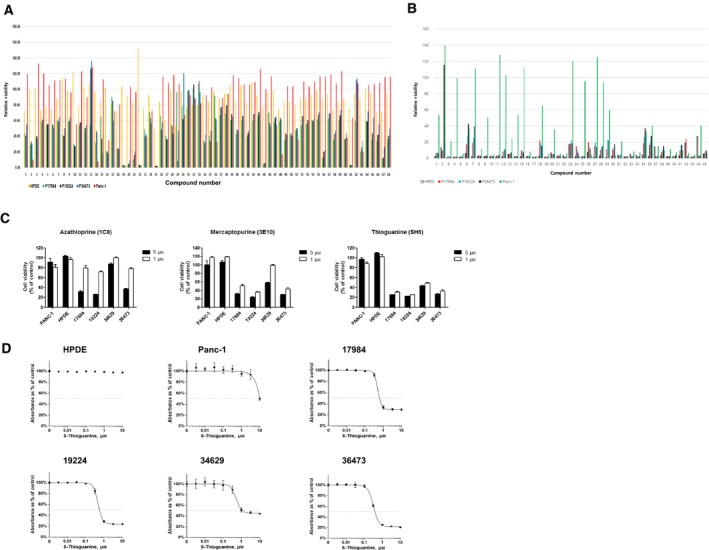
Identification of thiopurine compounds as PDAC‐specific drug candidates. (A) A graph showing cell viability after the treatment with 68 drug candidates (group A) of 4141 compounds from four libraries (see Methods). (B) A graph showing cell viability upon the treatment with 45 drug candidates (group B). The concentrations of compounds were 5 mm as a single point. (C) Two‐dose (1 and 5 mm) viability assay results on azathioprine (left), mercaptopurine (middle), and thioguanine (right). (D) IC_50_ curves of 6‐thioguanine for HPDE, Panc1, and four PDAC cells. Error bars indicate SEM.

Initial screening was performed on the four primary PDAC cells, Panc1 cell line, and HPDE. The overall scheme of the repositioning is presented in Fig. 1E, and assay procedures are summarized in Fig. 1F. Four compound libraries including the Selleckchem inhibitor library (1159 items), Prestwick chemical library (1200 items), LOPAC‐1280 active compound library, and the ENZO natural product library (502 items) were subjected to the screening. We identified 113 compounds that showed cytotoxicity (see Methods) towards at least three cancer cells at 5 μm, and 68 of them showed no or weaker cytotoxic effect toward HPDE cells (group A, Fig. 2A for the raw data, Table 1 for the list). The remaining 45 compounds that showed cytotoxicity toward both HPDE and cancer cells (group B, Fig. 2B for the raw data, Table 1 for the list).

**Table 1:** List of the compounds selected from the 1st screening

Chemical library			Natural product
Group A		Group B	
3‐Amino‐1‐propanesulfonic acid sodium	Paroxetine hydrochloride	AMG‐073 HCl (Cinacalcet HCl)	(–)‐Ouabain
Abitrexate (methotrexate)	Perhexiline maleate	AST‐1306	(β,β‐Dimethylacryl) Shikonin
Alexidine dihydrochloride	Perphenazine	Auranofin	Antibiotic A‐23187
APO866 (FK866)	Podophyllotoxin	BAY 11‐7082 (BAY 11‐7821)	Bufalin
Auranofin	Quinacrine dihydrochloride	CCT137690	C6 Ceramide
Azathioprine	Quinacrine dihydrochloride dihydrate	Celastrol	Celastrol
Azathioprine	Rottlerin	CI‐1033 (Canertinib)	Chelerythrine
AZD4547	S‐(+)‐Fluoxetine hydrochloride	Clomifene citrate (Serophene)	Cinobufagin
Bay 11‐7082	SB 242084 dihydrochloride hydrate	Daunorubicin hydrochloride	Citreoviridin
Benzethonium chloride	Sertraline hydrochloride	Digitoxigenin	Dehydrocostus lactone
beta‐Lapachone	SIB 1757	Digoxigenin	Deoxyshikonin
BIX 01294 trihydrochloride hydrate	Simvastatin	Digoxin	Digitoxin
Carprofen	Tamoxifen citrate	Dioscin (Collettiside III)	Ellipticine
Carvedilol	Terfenadine	Doxorubicin hydrochloride	Gambogic acid
Chicago sky blue 6B	Thiethylperazine dimalate	Fingolimod (FTY720)	Gliotoxin
CHM‐1 hydrate	Thimerosal	Foretinib (GSK1363089, XL880)	Mitomycin C
cis‐(Z)‐Flupenthixol dihydrochloride	Thioguanine	Idarubicin HCl	Oridonin
Clomipramine HCl (Anafranil)	Thioguanosine	IKK‐16	Parthenolide
Cycloheximide	Thioridazine hydrochloride	Ispinesib (SB‐715992)	Patulin
Dihydroouabain	Thioridazine hydrochloride	JTC‐801	Peruvoside
Duloxetine HCl (Cymbalta)	Trifluoperazine dihydrochloride	Lanatoside C	Plumbagin
Fiduxosin hydrochloride	Triflupromazine hydrochloride	LDN193189	Puromycin
Fluconazole	Vardenafil	LY2608204	Shikonin
Fluoxetine hydrochloride		Neratinib (HKI‐272)	Strophantidin
Fluphenazine dihydrochloride		NSC 95397	Thymoquinone
Fluspirilene		NSC348884 hydrate	Tubericidin
Fluvastatin sodium salt		NVP‐BGT226	Cimicifagoside
GR 127935 hydrochloride hydrate		OSU‐03012 (AR‐12)	Daidzein
GSK1070916		PHA‐665752	Oleanolic acid
Hexahydro‐sila‐difenidol hydrochloride		Ponatinib (AP24534)	Scopolin
Indatraline hydrochloride		PQ 401	Stigmasterol
L‐703,606 oxalate salt hydrate		Proscillaridin A	β‐chamigrenic acid
Maprotiline hydrochloride		Prothionamide	
Mercaptopurine		Sanguinarine chloride	
Mercaptopurine		SB 743921	
Methiothepin mesylate		Sertraline HCl	
Methylbenzethonium chloride		SRT1720	
Mibefradil dihydrochloride		Stattic	
MK‐2206 2HCl		Staurosporine	
MRS 2159		Thonzonium bromide	
NNC 55‐0396		Vortioxetine hydrobromide	
Nortriptyline hydrochloride		WP1066	
NS8593 hydrochloride		WP1130	
Palmitoyl‐DL‐Carnitine chloride		YM155	
Parbendazole		Zinc pyrithione	

### 3.3 Confirmation of the first hit reveals that purine analogs selectively kill PDAC cells

As a confirmation of the initial screening, we tested the 113 compounds at 1 and 5 μm in triplicate. We sought compounds that showed significant cytotoxicity at least toward three cancer cell lines at either 1 or 5 μm, but not toward HPDE cells (Figs S2–S4 for raw data on group A, obtained from three different chemical libraries). As a result, we selected three compounds (azathioprine, mercaptopurine, and 6‐thioguanine) that meet these conditions and all of them were identified as purine analogs. As shown in Fig. 2C, all three compounds manifested cancer cell‐specific toxicity except for azathioprine at 1 mm. This compound is an immunosuppressive medication that has been used for the treatment of multiple sclerosis (Confavreux and Moreau, 1996) or inflammatory bowel disease including Crohn's disease (Lamers *et al*., 1999). On the other hand, 6‐mercaptopurine is an anticancer agent (Skipper *et al*., 1954) that has been used against acute lymphocytic leukemia (ALL), chronic myeloid leukemia (CML), and Crohn's disease (Present, 1989). Similarly, 6‐thioguanine is also used against acute myeloid leukemia (AML), ALL, and CML (Ruutu and Elonen, 1991). These three compounds are in the same metabolic pathway previously described (Cara *et al*., 2004), suggesting that PDAC‐specific antitumor activity is closely related to purine biosynthesis. We next measured the IC_50_ values of the three compounds (Fig. 2D). In line with the previous screening results, we could not see death among HPDE cells at up to 10 μm 6‐TG treatment, whereas the primary PDAC cells showed IC_50_ ranging from 0.387 to 1.131 μm (Table 2). Mercaptopurine also exerted a selective cancer cell‐killing effect with one exception (cell clone 34629). Based on these results, we focused on 6‐TG for further analysis.

**Table 2:** IC_50_ values of the three thiopurine drug candidates

Name of drugs	IC_50_ value (μm)					
	HPDE	Panc‐1	17884	19224	34629	36473
Gemcitabine	0.816	> 10	0.18	0.145	0.258	0.049
Azathioprine	> 10	> 10	7.757	4.359	> 10	3.335
Mercaptopurine	> 10	> 10	1.345	1.119	> 10	1.232
6‐Thioguanine	> 10	9.943	0.622	0.562	1.131	0.387

### 3.4 Natural product drug candidates showed high cytotoxicity but failed to show cancer cell specificity

During the screening of a natural product library, the initial screening of 502 compounds yielded 32 hits (Table 1), and we examined 26 of them at 1‐ and 5‐μm doses (Fig. S5 for raw data). Notably, many of the candidates showed high toxicity at 1 μm, leading to dramatic cell death regardless of cell type. Therefore, we measured IC_50_ for 10 candidates showing high cytotoxicity, expecting some of them to exert a tumor‐specific killing effect. In contrast, as Fig. S6 shows, we could not find any compound that has superior cytotoxicity toward cancer cells (Table S2 for IC_50_ values).

### 3.5 TPMT, but not MTAP‐dependent 6‐TG susceptibility, explains cancer‐specific cytotoxicity

**Figure 3:**
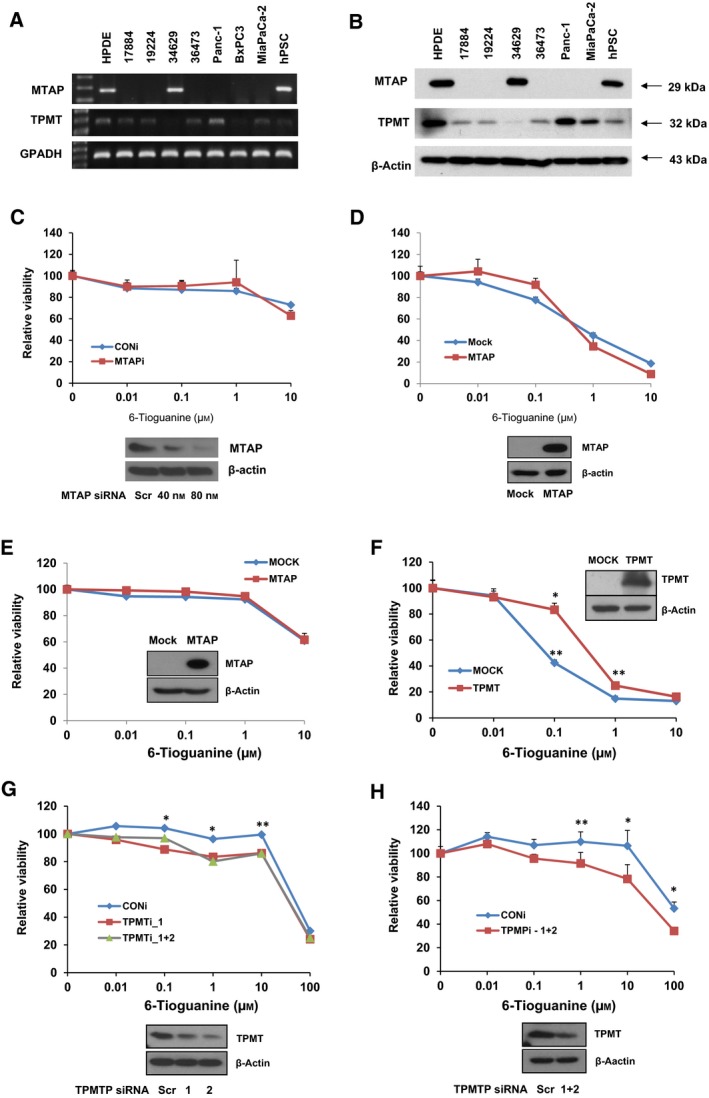
The expression of TPMT, rather than MTAP, affects the sensitivity of HPDE or PDAC cells to 6‐TG. (A,B) RT‐PCR (A) or Western blot (B) results of MTAP and TPMT for HPDE, primary PDAC, cancer cell lines, and stellate cells. The MTAP samples in (A) were run on the same gel. Intervening lanes were removed post‐acquisition as designated by the splice mark. (C) The knockdown effect of MTAP on the sensitivity of HPDE cells to 6‐TG. The lower panel shows the MTAP level in HPDE cells upon knockdown via siRNA. The upper panel contains a graph of relative viability in response to increasing doses of 6‐TG in control (blue) or MTAPi (red) cells. (D) The effect of MTAP overexpression in primary PDAC cells. The lower panel shows MTAP levels in MTAP^low^ 17884 cells. The upper panel present a graph of relative viability in response to increasing doses of 6‐TG in control (Mock, blue) or MTAP‐overexpressing cells (red). (E) Viability assay results obtained from Panc1 cells. The WB panel shows MTAP expression and the graph presents viability in response to 6‐TG in control (Mock, blue) or MTAP‐overexpressing Panc1 cells (red). (F) The effect of TPMT overexpression on the viability of primary PDAC cells. The graph shows relative viability in response to increasing doses of 6‐TG in control (Mock, blue) or TPMT‐overexpressing cells (red). The upper right panel shows TPMT expression, by WB. (G,H) TPMT affects the sensitivity of HPDE (F) or PDAC (G) cells to 6‐TG. Western blots show the level of TPMT in control or TPMT siRNA‐treated cells. The graphs present relative viability in response to increasing doses of 6‐TG in control (blue) or TPMTi (green or red) cells. Error bars indicate SEM.

As our drug‐repositioning results strongly suggested thiopurines inhibit primary PDAC cell growth more efficiently compared with normal HPDE, we questioned how 6‐TG, a thiopurine, implements its tumor cell‐specific killing effect. One report showed that MTAP abrogates phosphoribosylation conversion of 6‐TG to a toxic derivative (Munshi *et al*., 2014). Interestingly, another report revealed that the *p16* gene is often lost along with the *MTAP* gene in pancreatic cancer (Lubin and Lubin, 2009; Munshi *et al*., 2014). RT‐PCR and Western blot analysis indicated that MTAP was expressed in HPDE and 34 629 cells (which showed the highest IC_50_ among four PDAC cell lines; Fig. 3A,B). Therefore, we first tested whether the expression of *MTAP* can confer resistance to 6‐TG‐induced toxicity. The transfection of *MTAP* small interfering RNA (siRNA) into HPDE cells showed an effective knockdown of MTAP (Fig. 3C) but did not affect sensitivity to 6‐TG (Fig. 3C, P = 0.668, two‐way ANOVA test). We also tested the overexpression of MTAP in the 17 884 cell line, which had low MTAP expression (Fig. 3A,B). In this case, we observed significant resistance at low concentrations of 6‐TG (*P* = 0.03 at 0.01 μm and 0.005 at 0.1 μm), but this was reversed at high 6‐TG concentrations (Fig. 3D). Therefore, we concluded that 6‐TG overexpression does not consistently confer resistance to 6‐TG. This result was also confirmed in Panc1 cells (Fig. 3E, P = 0.771, two‐way ANOVA test).

We therefore searched for another possible mechanism underlying 6‐TG resistance and focused on *TPMT*. A defect or decreased level of this enzyme results in decreased methylation of a thiopurine drug, which increases its toxicity (Krynetski and Evans, 2003). Western blot and real‐time PCR analysis showed high TPMT expression in HPDE and Panc1 cell lines, both of which had high IC_50_ values toward 6‐TG (Fig. 3A,B, Fig. S7, Table S2). Consistently, we found overexpression of TPMT conferred resistance to 6‐TG‐induced toxicity (Fig. 3F). Moreover, a knockdown of *TPMT* in both TPMT‐high HPDE and Panc1 cells sensitized them to 6‐TG, at 0.1–10 μm for HPDE and 1–100 μm for Panc1 cells, respectively (Fig. 3G,H). These results collectively suggested that the *TPMT* expression level affects efficacy of 6‐TG against cancer cells.

### 3.6 6‐TG inhibits the BRAF‐MEK‐ERK pathway and induces apoptotic cell death in a cancer cell‐specific manner

**Figure 4:**
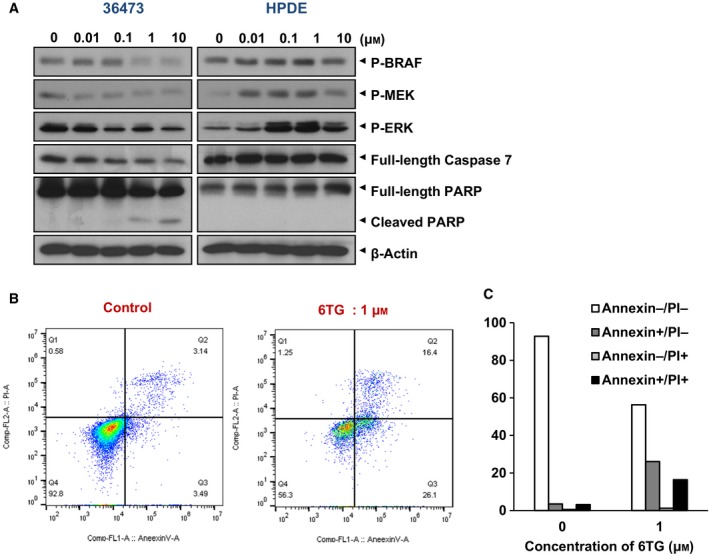
6‐TG inhibits the Ras‐Raf‐MAPK signaling cascade and induces apoptosis in PDAC cells. (A) Western blot analysis of cells for p‐BRAF, p‐MEK, p‐ERK, caspase 7, and PARP in PDAC (36 473) or HPDE cells after the various doses of 6‐TG treatment. β‐Actin served as a loading control. (B) Apoptosis assay measured by Annexin V/PI staining. After treatment of 6‐TG for 48 h, cells were analyzed by flow cytometry. (C) The percentage of annexin V/PI‐positive cells from (B) is shown as a graph.

As we confirmed that 6‐TG inhibits PDAC cell proliferation, we next examined the molecular alterations specifically triggered by 6‐TG in cancer cells. Because 6‐TG has been shown to regulate GTPase activity (de Boer *et al*., 2007), we analyzed the effect of 6‐TG on the RAS‐RAF‐MAP Kinase pathway that is frequently activated in PDAC. The results in Fig. 4A indicate that the phosphorylated (activated) levels of BRAF, MEK, and ERK were decreased in primary PDAC cell line 36 473 by the 6‐TG treatment (left panels), whereas HPDE cells were not affected (right panels). In addition, we observed a reduced level of full‐length Caspase 7 as well as increased PARP cleavage in the 36 473 cells but not in the HPDE, suggesting that 6‐TG triggers apoptosis in a cancer cell‐specific manner. We confirmed the apoptotic effect of 6TG by annexin V/PI staining, showing the 6TG treatment induced increased apoptosis compared with the control cells (Fig. 4B,C).

### 3.7 6‐TG has an antitumor effect in combination with gemcitabine in a patient‐derived xenograft model

**Figure 5:**
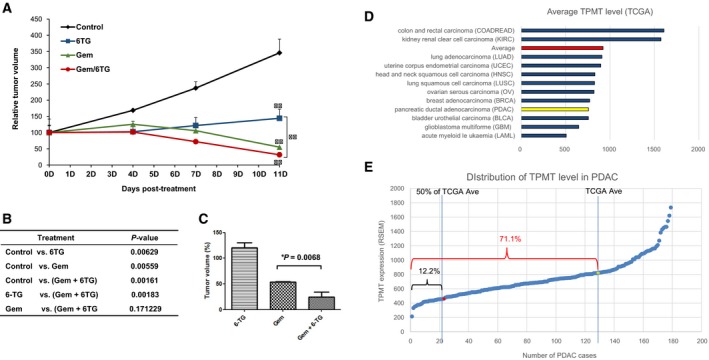
*In vivo* efficacy of 6‐thioguanine in the pancreatic patient‐derived xenograft model. (A) 6‐TG or gemcitabine was administered as monotherapy or in combination, and tumor growth was monitored (*n* = 20). Error bar indicates SEM. (B) Summary of statistical analysis of the tumor volumes during drug treatment. A significant difference was observed between gemcitabine (single) and gemcitabine plus 6‐TG (*P* < 0.0001). Likewise, gemcitabine plus 6‐TG showed a significantly better antitumor effect than 6‐TG alone (*P* = 0.01). (C) Graph showing the average volume of the tumors at the endpoint. Note a significant decrease of tumor volume in the gemcitabine plus 6‐TG group, as compared with gemcitabine alone. Error bar indicates SEM. (D) Bar chart showing the average level of TPMT for various types of tumor, obtained from TCGA data. Red bar indicates average of all tumor types and yellow bar shows TPMT level in PDAC. (E) Estimation of TPMT‐low portion in PDAC cases. A total of 180 cases are analyzed from TCGA data and plotted in ascending order. The line on the right indicates the cutoff of average TPMT level from all types of tumor analyzed in (D). Another line on the left shows 50% of TCGA average.

To validate the antitumor effect of 6‐TG *in vivo*, we introduced a patient‐derived xenograft model of PDAC (Jung *et al*., 2016). Previously characterized 19 224 patient‐derived xenograft (PDX) cells lack P53 expression and express SMAD4 weakly, but showed high phospho (p)‐ERK and p‐AKT levels. We selected this PDX because the matching PDAC cells (19 224) showed a good response to 6‐TG, and TPMT expression was relatively low (Fig. 3A,B). In addition to 6‐TG monotherapy, a combination of 6‐TG with gemcitabine was tested, based on the *in vitro* data in PDAC cells (Fig. S8). Tumor volume data presented in Fig. 5A indicated that 6‐TG treatment alone (marked as blue rectangles) showed significantly suppressed but stationary tumor growth (Fig. 5B, see Discussion). In contrast, gemcitabine, a common chemotherapeutic agent for PDAC, manifested a tumor cell‐killing effect (Fig. 5A, marked as green triangles and B for statistical value). Importantly, when 6‐TG was used in combination with gemcitabine, we observed a significantly reduced tumor growth compared with 6‐TG alone (red circles, *P* < 0.005). The tumor volume at the final time point supported this finding (Fig. 5C). Based on the result showing antitumor effect of 6‐TG in TPMT‐low PDAC, we then analyzed TCGA data to estimate the expression level of TPMT in PDAC compared with various types of cancer. We also questioned how large a portion of PDAC is TPMT‐low. The results are shown in Fig. 5D,E. Among 12 cancer types analyzed, PDAC was 9th, suggesting the 6‐TG can be relatively effective in PDAC. Indeed, Fig. 5E shows 71.1% of the PDAC has a TPMT level lower than the TCGA average. If we apply a more stringent cutoff, then we see that 12.2% of PDAC shows a TPMT level less than 50% of the TCGA average. We speculate that these populations could represent a beneficial group using the 6‐TG treatment. Altogether, these data suggested that 6‐TG is an effective antitumor agent in combination with gemcitabine *in vivo*, for the tumors with low expression of *TPMT*.

## 4 Discussion

6‐TG was initially reported in 1955 (Garattini and Mussini, 1955) and is currently used against inflammatory bowel disease (Mantzaris, 2017) as well as several lymphoid tumors such as AML, ALL, and CML (Karran and Attard, 2008). Once 6‐TG enters the cell, it is converted to 6‐thioguanine nucleotide (6‐TN), which is toxic (Ishiguro *et al*., 1984). Its toxicity is mainly caused by the incorporation of 6‐TN into DNA during the S phase of the cell cycle (Lennard *et al*., 1989), thereby indicating its effectiveness in cancer. Alternatively, it can inactivate a GTP‐binding protein such as Rac1 (Tiede *et al*., 2003). Considering that PDAC is associated with a frequent *KRas* activating mutation, further research is needed to clarify whether 6‐TG can inhibit mutant *KRas* signaling.

Previously, a phase II clinical trial of 6‐TG involving 32 PDAC patients was reported, but it showed no significant therapeutic effect (Ajani *et al*., 1991). We speculate that this is mainly due to the desmoplastic microenvironment of PDAC, which usually interferes with drug delivery into cancer cells (Erkan, 2013; Lunardi *et al*., 2014). When we tested mouse stromal content in PDX model using PTGER2 primer (Alcoser *et al*., 2011), we found that it represented 10–15% of mouse cells (Fig. S9). Consequently, we found 6‐TG treatment alone in the PDX model did not show tumor regression (Fig. 5A, blue line), whereas *in vitro* testing showed an efficient cytotoxic effect (Fig. 2D). Therefore, along with the identification of a therapeutic agent in PDAC, there is an urgent need for an effective delivery method. In this regard, recent approaches including nanoparticles, enzymatic treatment, and combination with stellate cell targeting agent deserve further attention (Wang *et al*., 2017). In our study, we found *TPMT* expression to be lower in cancer cells than in normal HPDE cells. By far, the regulatory mechanism of *TPMT* expression is not clearly understood. In 1997, Krynetski *et al*. isolated the *TPMT* gene promoter, finding 71% GC content without a TATA box or CCAAT elements. By contrast, polymorphism of the *TPMT* gene has been characterized extensively because it determines patient adverse effects due to toxicity caused by the loss of functional *TPMT* variants (Tamm *et al*., 2017). The regulation of *TPMT* expression by transcription factors and/or epigenetic modification will aid researchers to apply 6‐TG precisely to a subgroup of PDAC patients.

During the screening of the natural product library, we found that each of the candidate compounds had a high cytotoxicity. In our primary hit list (Table 1), we found that thymoquinone was reported previously as an anti‐PDAC agent (Relles *et al*., 2016). In contrast, our subsequent experiment (Fig. S5 and 5C8) showed that this compound kills HPDE cells better than cancer cells. Some studies also identified diindolylmethane as a candidate agent for PDAC (Li *et al*., 2013), but it was not effective in our study. We can speculate that the physiological (as present in its natural source) level of each natural compound is much lower than what we tested, and furthermore, its effect may be stronger in combination with multiple ingredients of other natural products.

## 5 Conclusions

Our results presented here strongly suggest that 6‐TG is a PDAC cell‐specific antitumor agent. Further study including modification for better efficacy and the development of an efficient delivery method will facilitate the application of this drug in clinical practice.

## Author contributions

IK wrote the manuscript and supervised drug screening. YSC wrote the manuscript and performed the MTAP experiments. JHS helped drug screening and prepared part of the figures. EAC performed the TPMT experiments. SP mainly performed the HTS screening. EJL helped primary cell culture. SCK provided clinical information and supported funding. SC conceived the main idea and wrote the manuscript.

## Supporting information


**Fig. S1.** RT‐PCR results on extracellular‐matrix genes from HPDE, PDAC, and stellate cells.
**Fig. S2.** Confirmation of A‐group candidates obtained from the Prestwick chemical library.
**Fig. S3.** Confirmation of A‐group candidates obtained from the Selleckchem chemical library.
**Fig. S4.** Confirmation of A‐group candidates obtained from the LOPAC chemical library.
**Fig. S5.** Confirmation for the cytotoxicity of drug candidates obtained from the Natural product library.
**Fig. S6.** Measurement of IC_50_ values for the selected 10 compounds from the Natural product library.
**Fig. S7.** Proliferation assay results showing the growth inhibitory effect of 6TG, gemcitabine, and their combination in pancreatic cancer primary cells.
**Fig. S8.** RNA levels of TPMT was analyzed by real‐time PCR.
**Fig. S9.** Measurement of mouse cell content in PDX, using human/mouse PTGER2 primers.
**Table S1.** Clinicopathological parameters of pancreatic cancer patients for primary cell culture.
**Table S2.** IC_50_ values of the natural product drug candidates.
