# Supplementary material for: A drug‐repositioning screen for primary pancreatic ductal adenocarcinoma cells identifies 6‐thioguanine as an effective therapeutic agent for TPMT‐low cancer cells
Source: Mol Oncol. 2018 Aug 29;12(9):1526–39. doi: 10.1002/1878-0261.12364 (PMC6120251; doi:10.1002/1878-0261.12364)
Supplement: Supplementary file 1 — Fig. S1. RT‐PCR results on extracellular‐matrix genes from HPDE, PDAC, and stellate cells. Fig. S2. Confirmation of A‐group candidates obtained from the Prestwick chemical library. Fig. S3. Confirmation of A‐group candidates obtained from the Selleckchem chemical library. Fig. S4. Confirmation of A‐group candidates obtained from the LOPAC chemical library. Fig. S5. Confirmation for the cytotoxicity of drug candidates obtained from the Natural product library. Fig. S6. Measurement of IC50 values for the selected 10 compounds from the Natural product library. Fig. S7. Proliferation assay results showing the growth inhibitory effect of 6TG, gemcitabine, and their combination in pancreatic cancer primary cells. Fig. S8. RNA levels of TPMT was analyzed by real‐time PCR. Fig. S9. Measurement of mouse cell content in PDX, using human/mouse PTGER2 primers. Table S1. Clinicopathological parameters of pancreatic cancer patients for primary cell culture. Table S2. IC50 values of the natural product drug candidates. [file MOL2-12-1526-s001.pptx]

## Slide 1
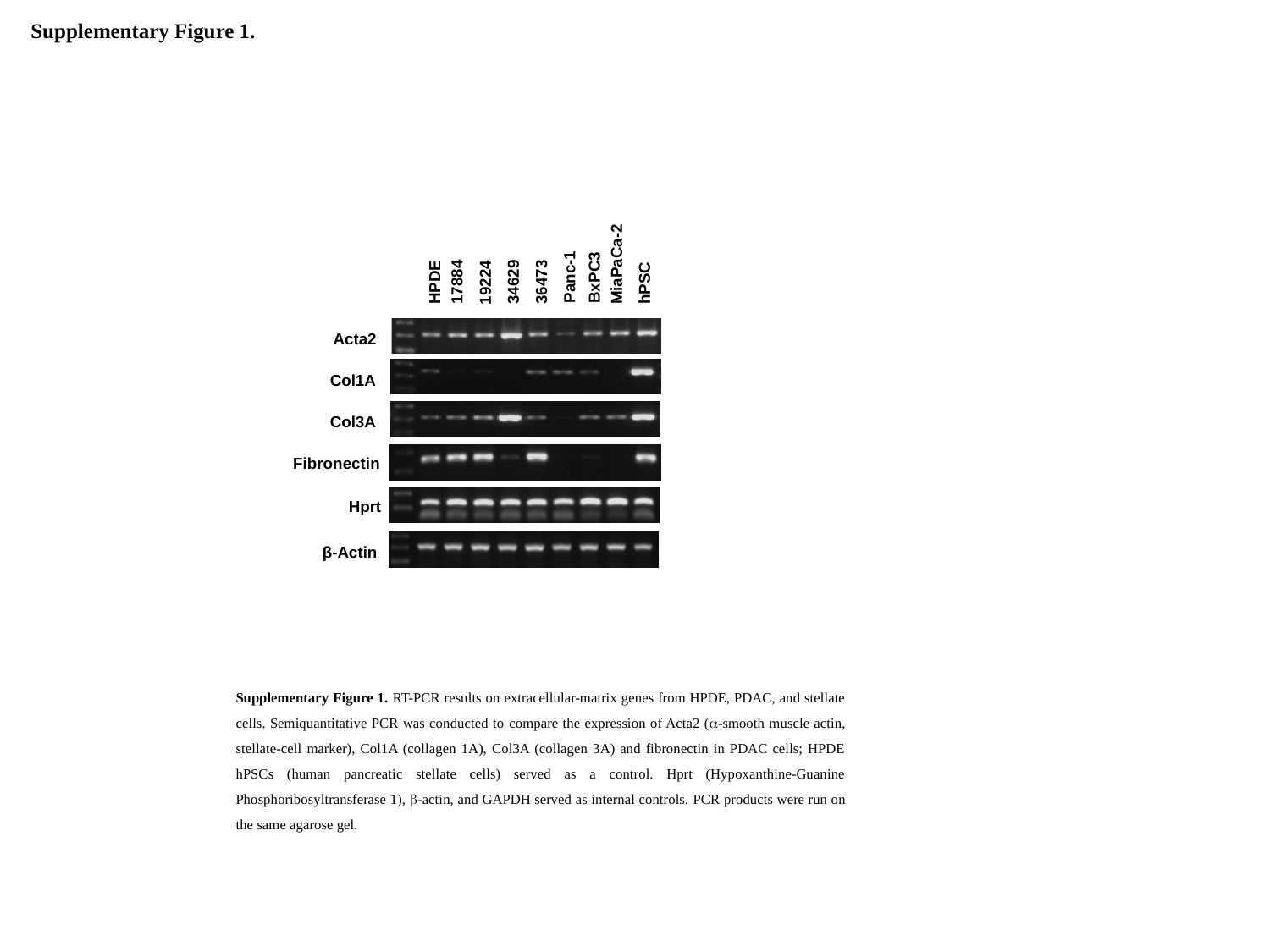

Supplementary Figure 1.
MiaPaCa-2
Panc-1
BxPC3
HPDE
17884
34629
36473
hPSC
19224
Acta2
Col1A
Col3A
Fibronectin
Hprt
β-Actin
Supplementary Figure 1. RT-PCR results on extracellular-matrix genes from HPDE, PDAC, and stellate cells. Semiquantitative PCR was conducted to compare the expression of Acta2 (α-smooth muscle actin, stellate-cell marker), Col1A (collagen 1A), Col3A (collagen 3A) and fibronectin in PDAC cells; HPDE hPSCs (human pancreatic stellate cells) served as a control. Hprt (Hypoxanthine-Guanine Phosphoribosyltransferase 1), b-actin, and GAPDH served as internal controls. PCR products were run on the same agarose gel.

## Slide 2
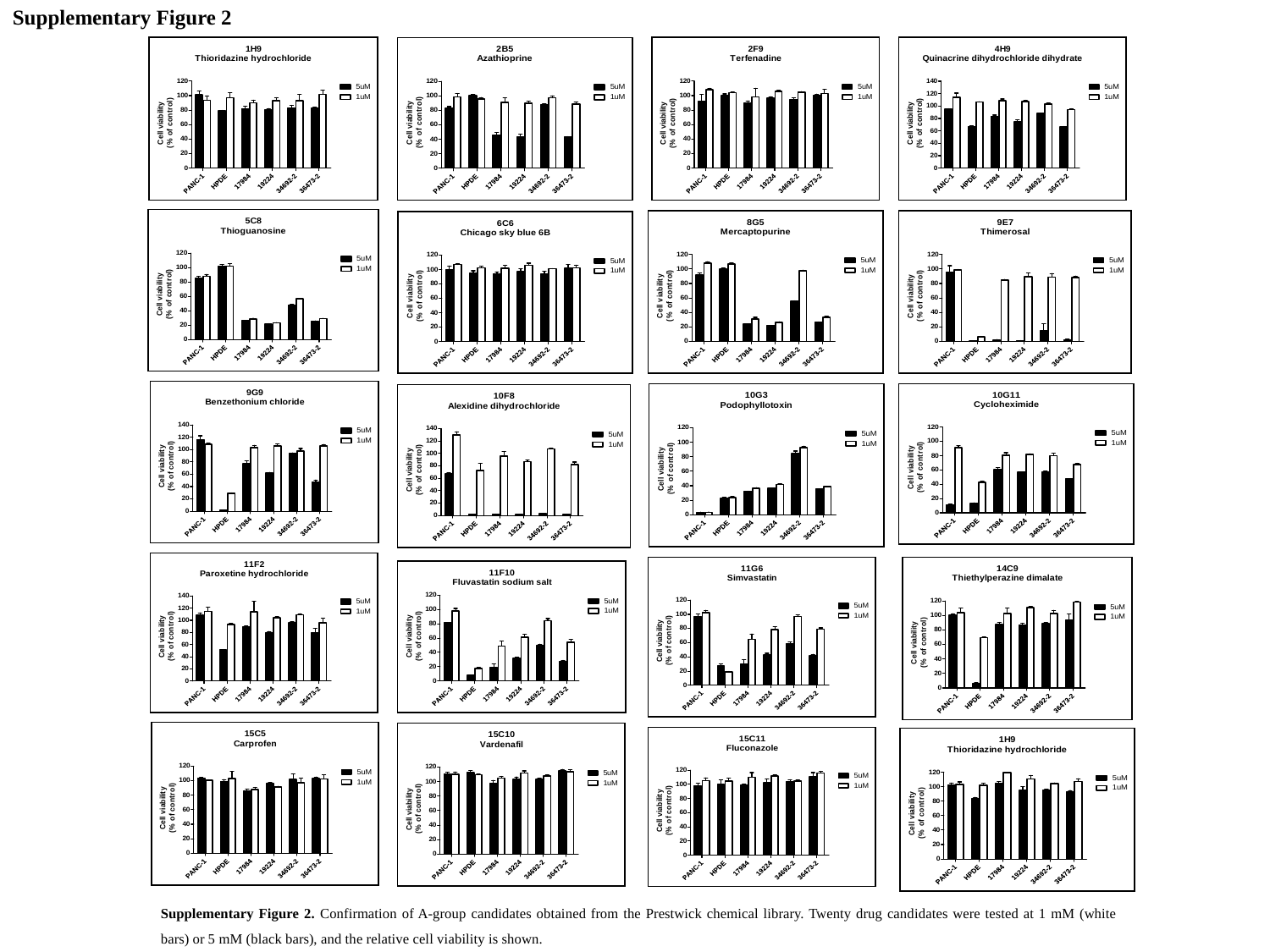

Supplementary Figure 2
Supplementary Figure 2. Confirmation of A-group candidates obtained from the Prestwick chemical library. Twenty drug candidates were tested at 1 mM (white bars) or 5 mM (black bars), and the relative cell viability is shown.

## Slide 3
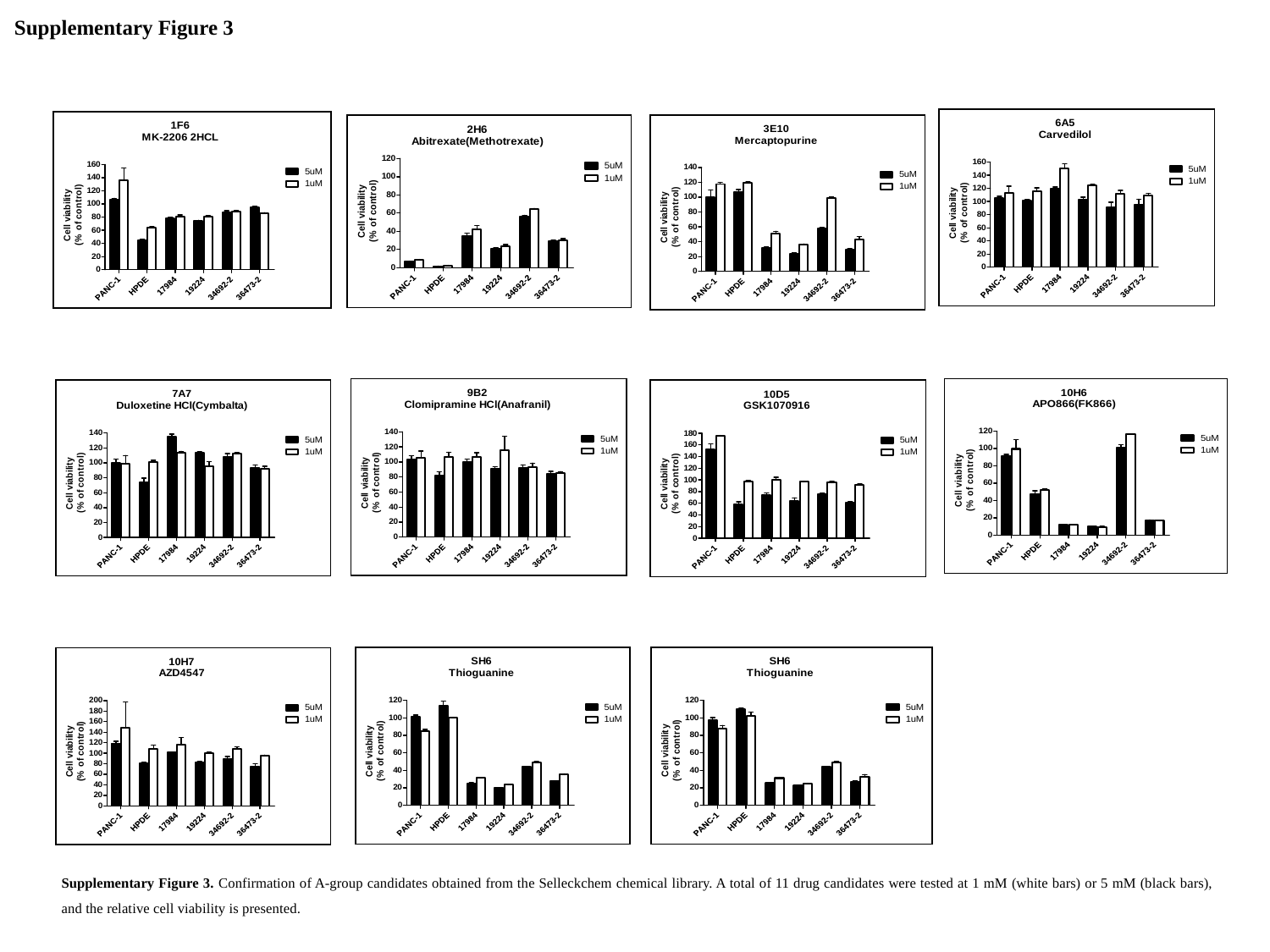

Supplementary Figure 3
Supplementary Figure 3. Confirmation of A-group candidates obtained from the Selleckchem chemical library. A total of 11 drug candidates were tested at 1 mM (white bars) or 5 mM (black bars), and the relative cell viability is presented.

## Slide 4
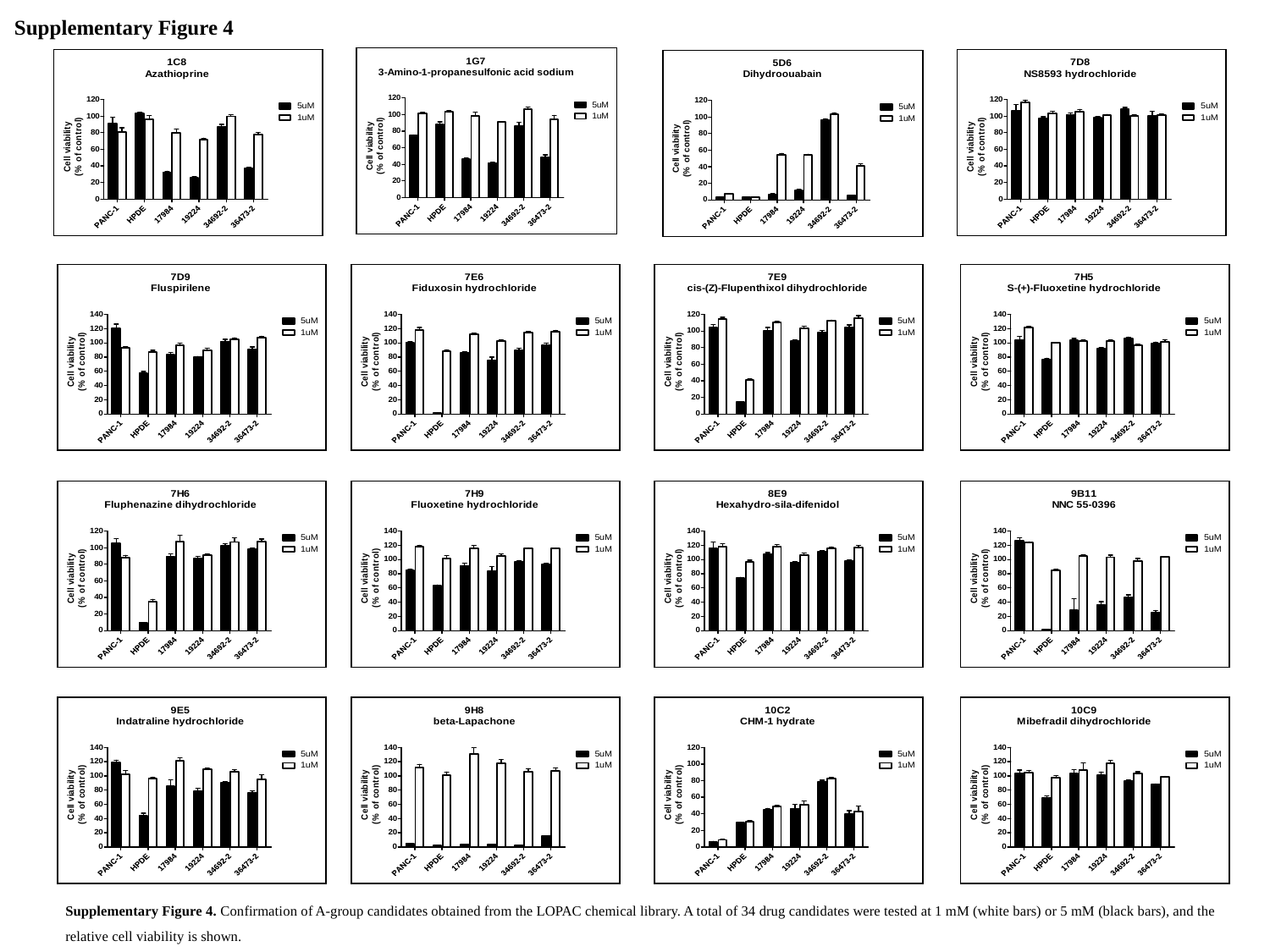

Supplementary Figure 4
Supplementary Figure 4. Confirmation of A-group candidates obtained from the LOPAC chemical library. A total of 34 drug candidates were tested at 1 mM (white bars) or 5 mM (black bars), and the relative cell viability is shown.

## Slide 5
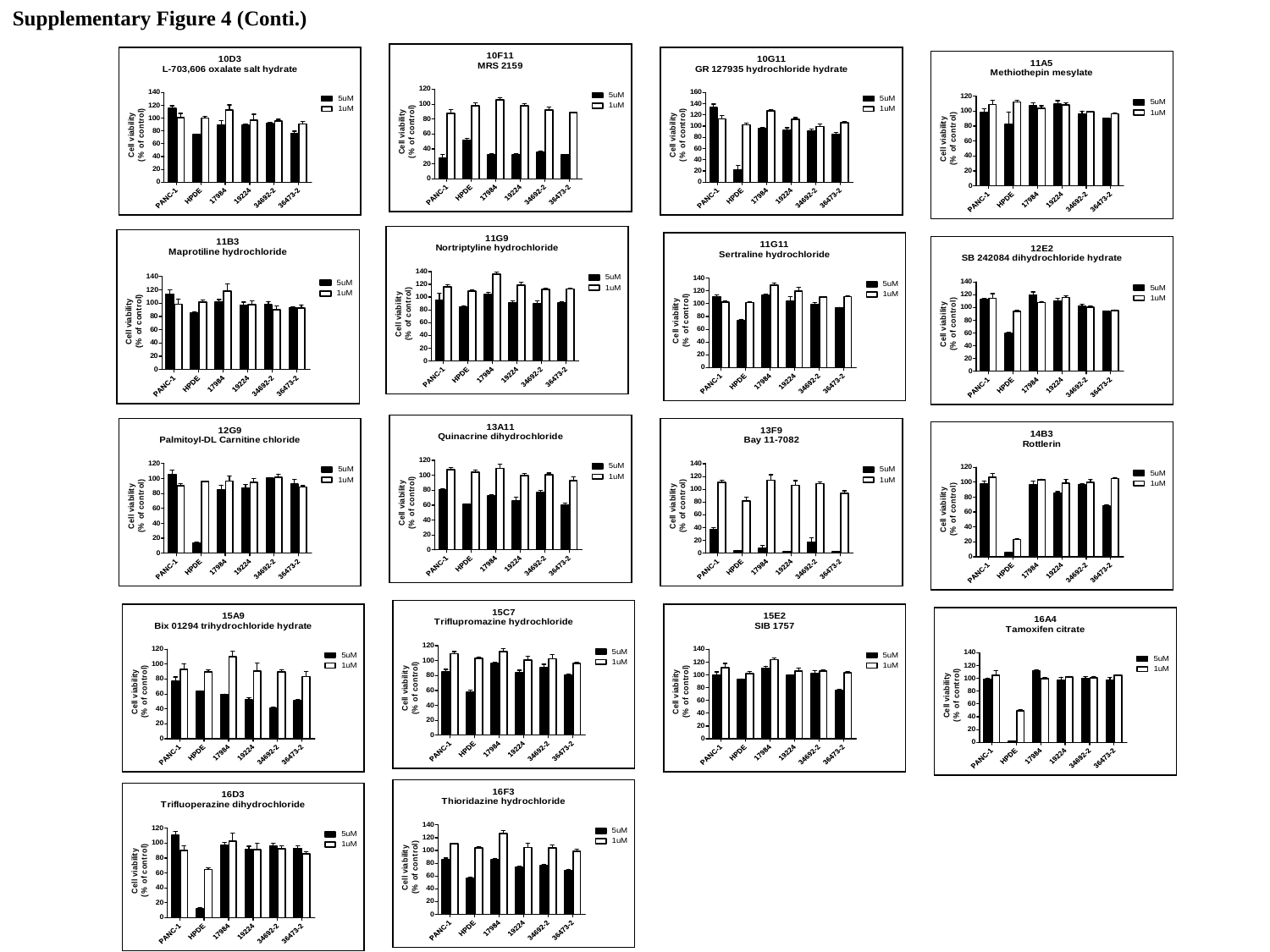

Supplementary Figure 4 (Conti.)

## Slide 6
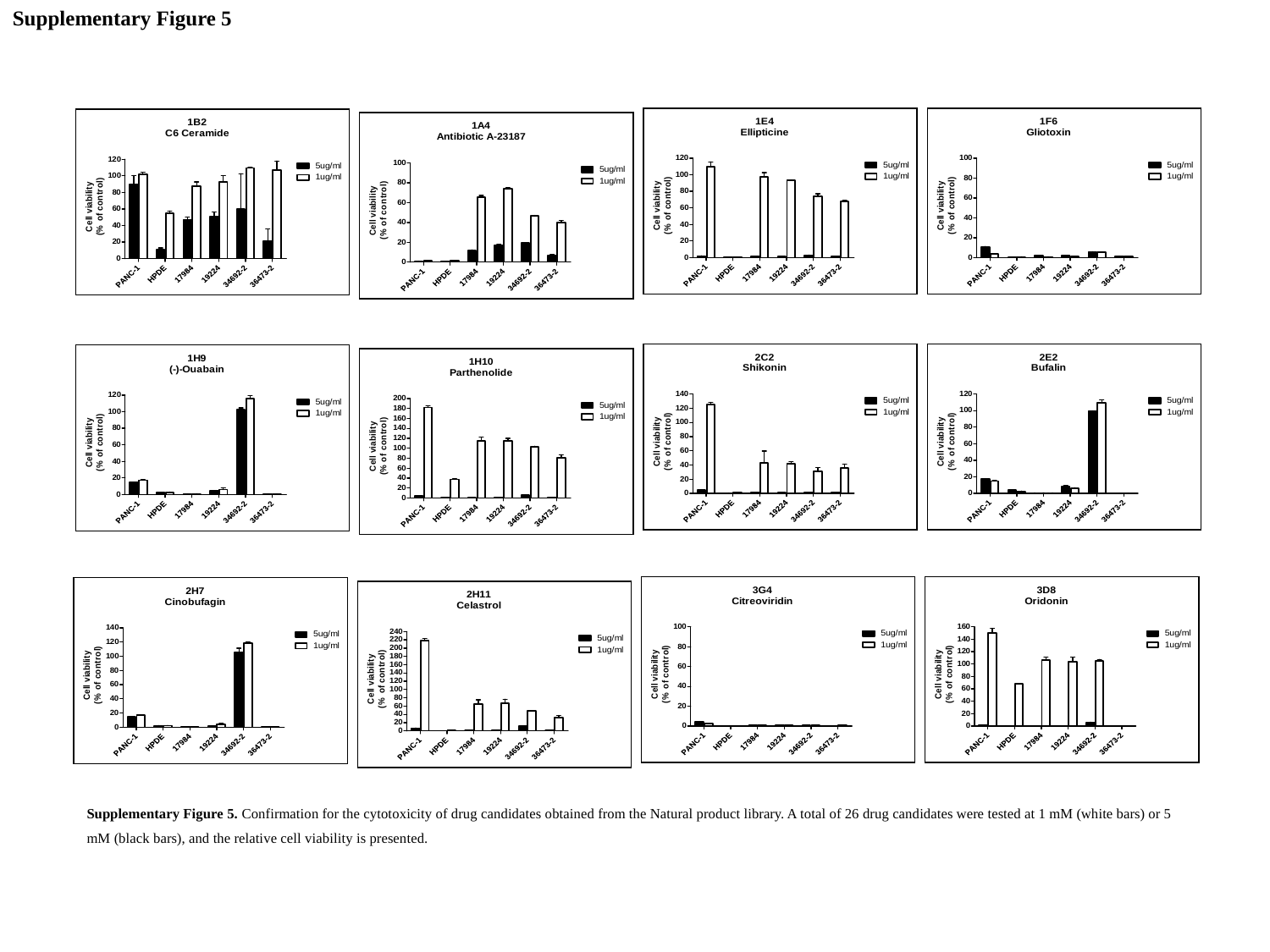

Supplementary Figure 5
Supplementary Figure 5. Confirmation for the cytotoxicity of drug candidates obtained from the Natural product library. A total of 26 drug candidates were tested at 1 mM (white bars) or 5 mM (black bars), and the relative cell viability is presented.

## Slide 7
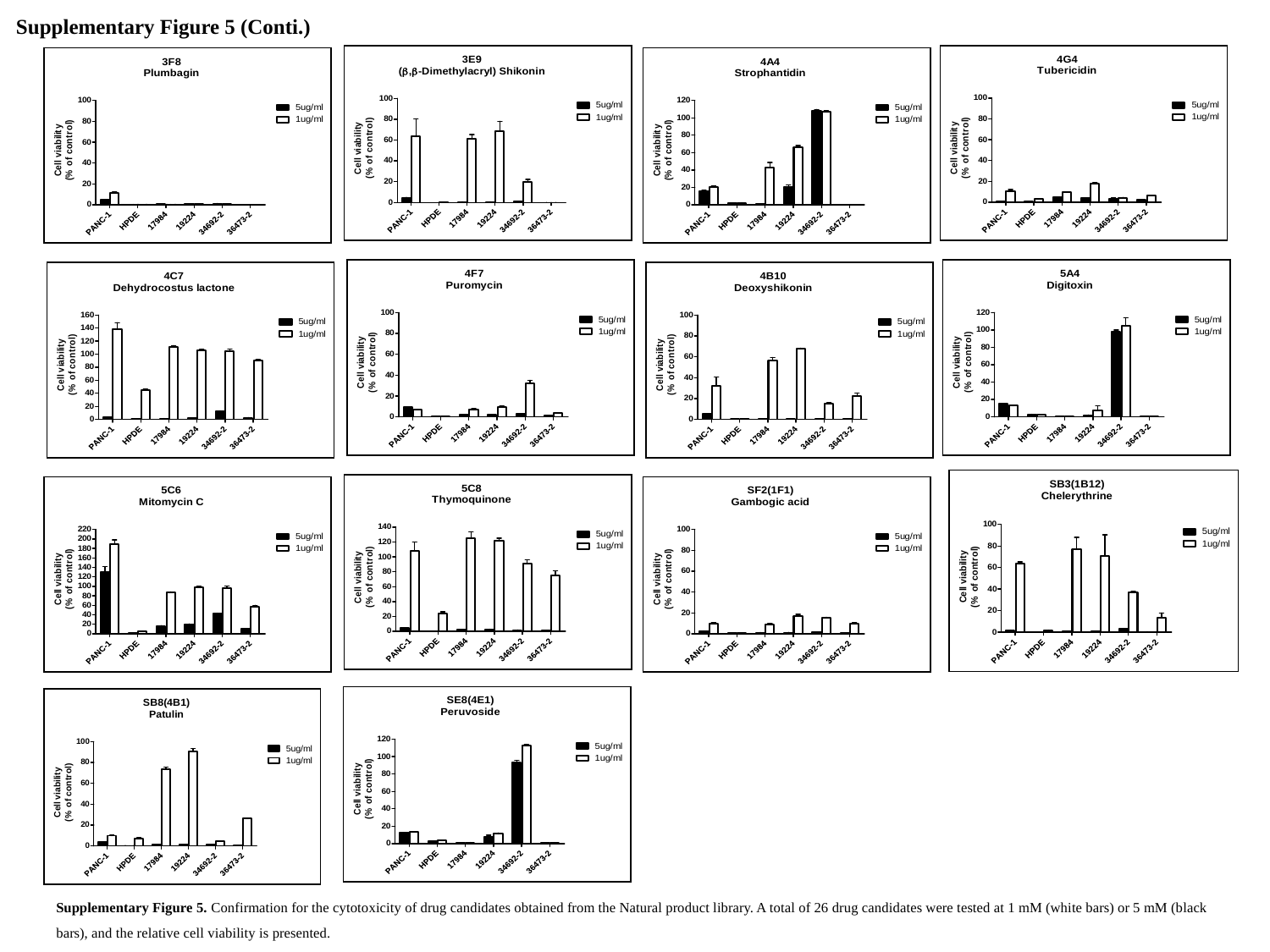

Supplementary Figure 5 (Conti.)
Supplementary Figure 5. Confirmation for the cytotoxicity of drug candidates obtained from the Natural product library. A total of 26 drug candidates were tested at 1 mM (white bars) or 5 mM (black bars), and the relative cell viability is presented.

## Slide 8
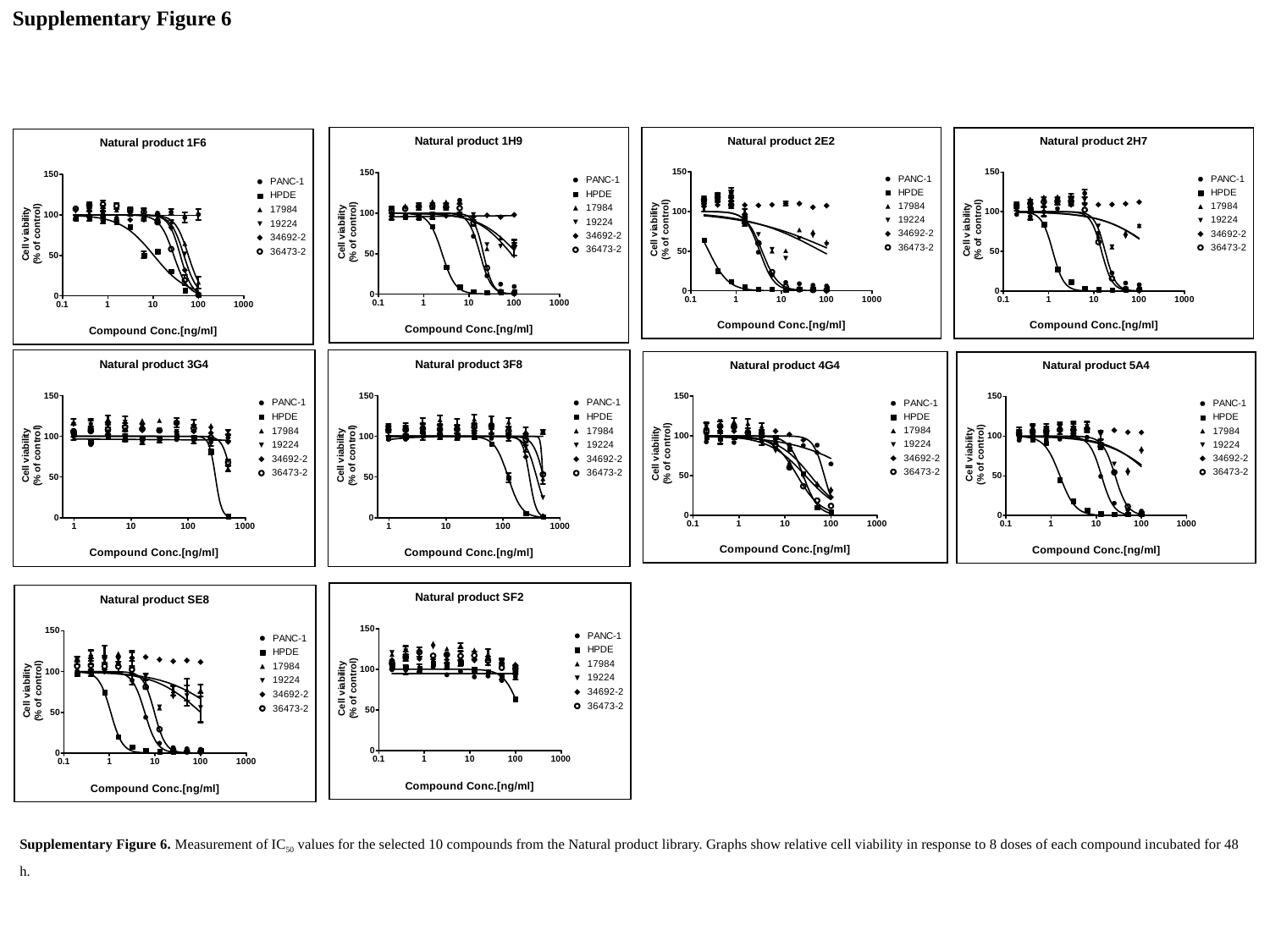

Supplementary Figure 6
Supplementary Figure 6. Measurement of IC50 values for the selected 10 compounds from the Natural product library. Graphs show relative cell viability in response to 8 doses of each compound incubated for 48 h.

## Slide 9
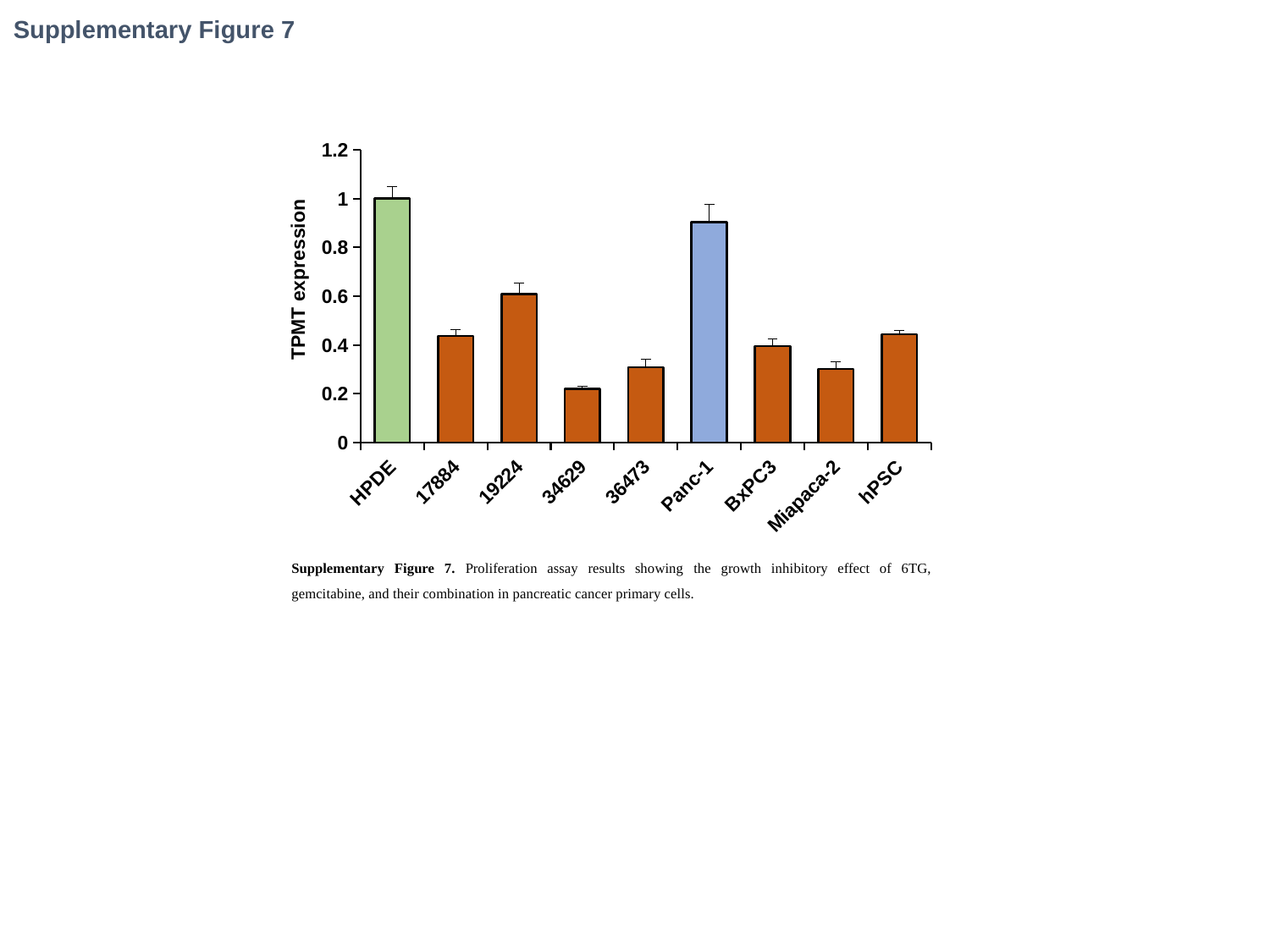

Supplementary Figure 7
### Chart
| Category | |
|---|---|
| HPDE | 0.9999999999999999 |
| 17884 | 0.43706794016534645 |
| 19224 | 0.6085130005490472 |
| 34629 | 0.22013584259409244 |
| 36473 | 0.30922899972958356 |
| Panc-1 | 0.9023968696731726 |
| BxPC3 | 0.39588502709534246 |
| Miapaca-2 | 0.3015906766237078 |
| hPSC | 0.4433481206868431 |TPMT expression
Supplementary Figure 7. Proliferation assay results showing the growth inhibitory effect of 6TG, gemcitabine, and their combination in pancreatic cancer primary cells.

## Slide 10
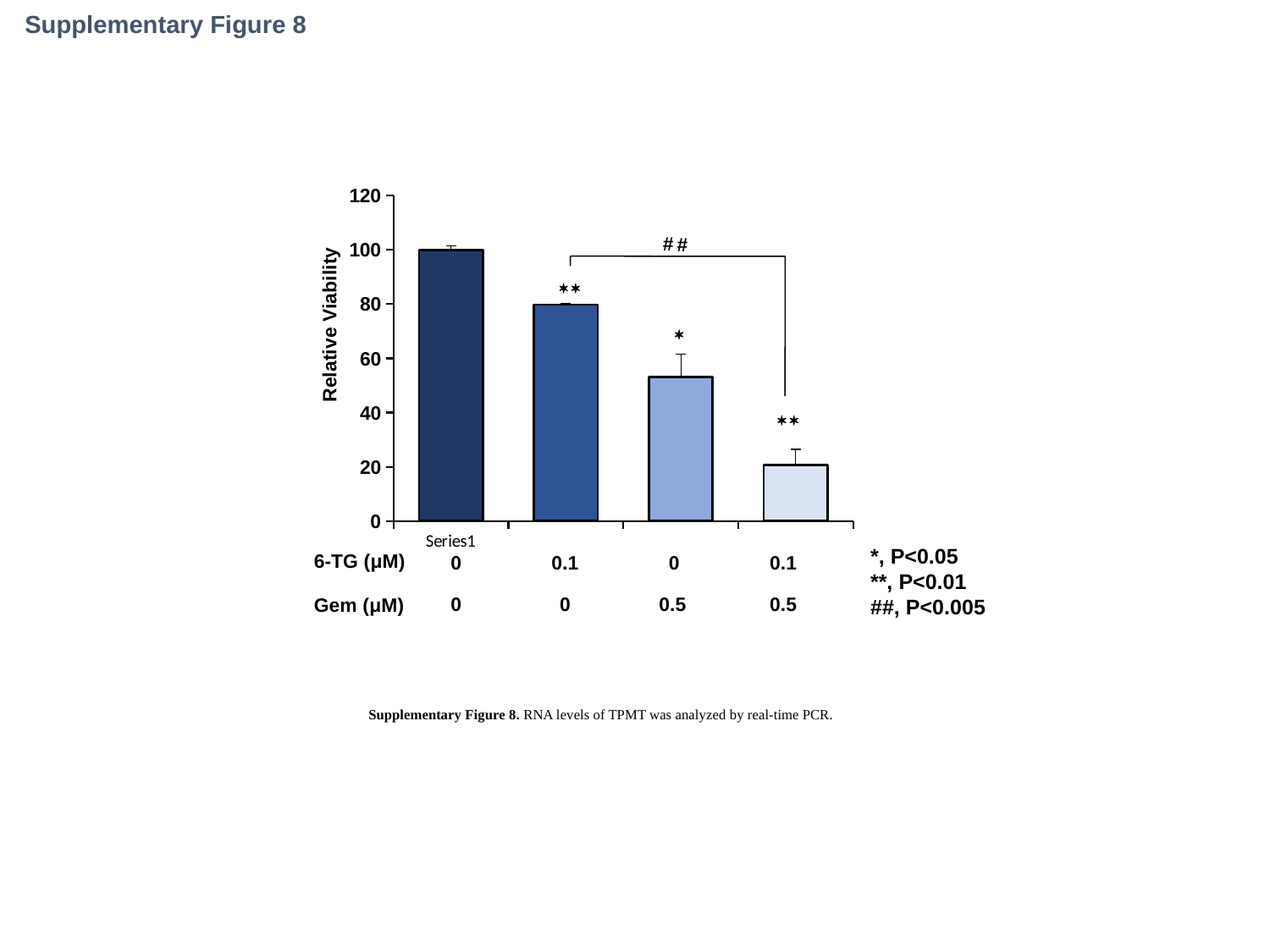

Supplementary Figure 8
### Chart
| Category | |
|---|---|#
#
Relative Viability





*, P<0.05
**, P<0.01
##, P<0.005
6-TG (μM)
0
0.1
0
0.1
0
0
0.5
0.5
Gem (μM)
Supplementary Figure 8. RNA levels of TPMT was analyzed by real-time PCR.

## Slide 11
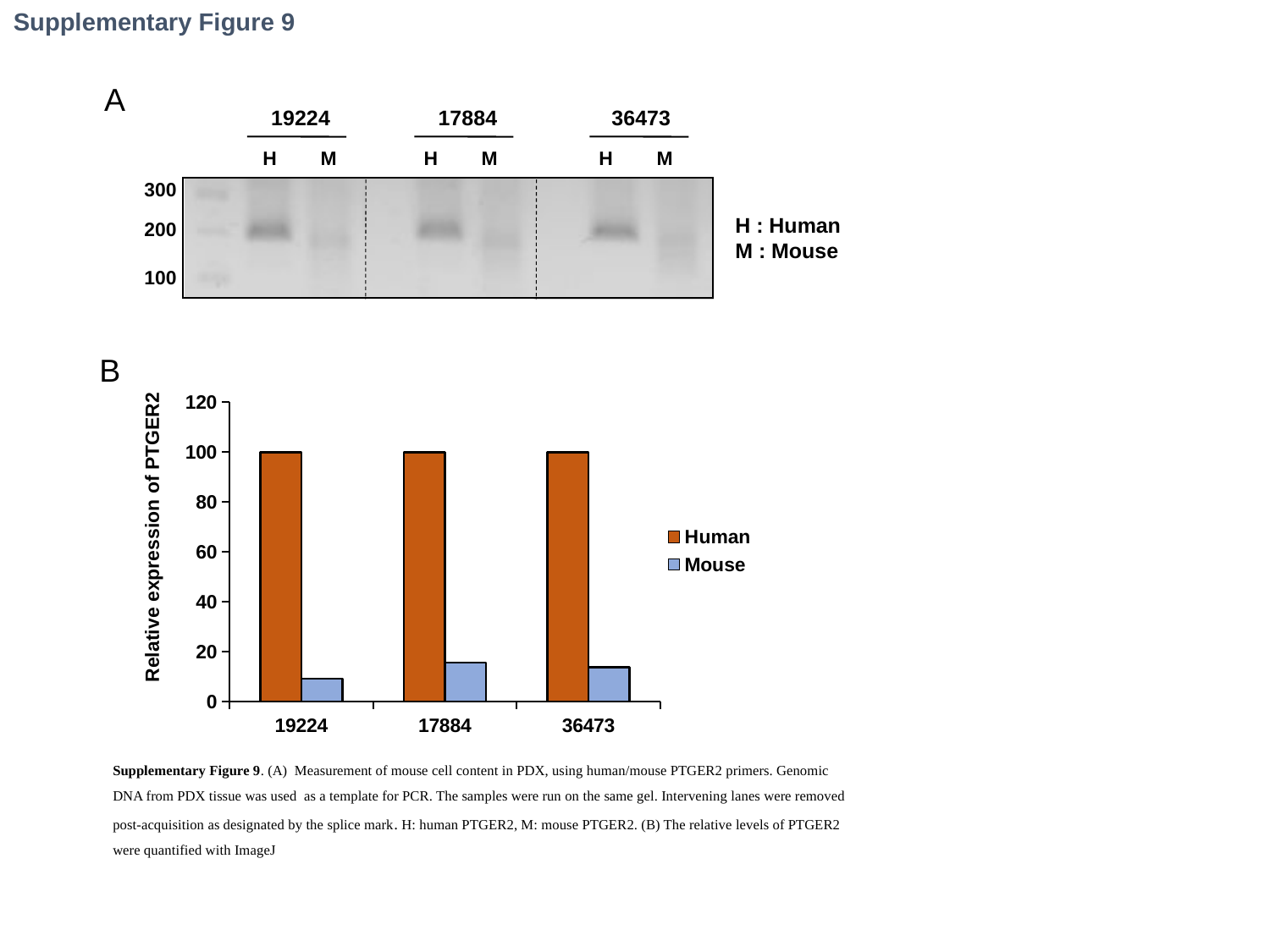

Supplementary Figure 9
A
19224
17884
36473
H
M
H
M
H
M
300
H : Human
M : Mouse
200
100
B
### Chart
| Category | Human | Mouse |
|---|---|---|
| 19224 | 100.0 | 9.140271493212655 |
| 17884 | 100.0 | 15.514805096964729 |
| 36473 | 100.0 | 13.621040812690214 |Relative expression of PTGER2
Supplementary Figure 9. (A) Measurement of mouse cell content in PDX, using human/mouse PTGER2 primers. Genomic DNA from PDX tissue was used as a template for PCR. The samples were run on the same gel. Intervening lanes were removed post-acquisition as designated by the splice mark. H: human PTGER2, M: mouse PTGER2. (B) The relative levels of PTGER2 were quantified with ImageJ

## Slide 12
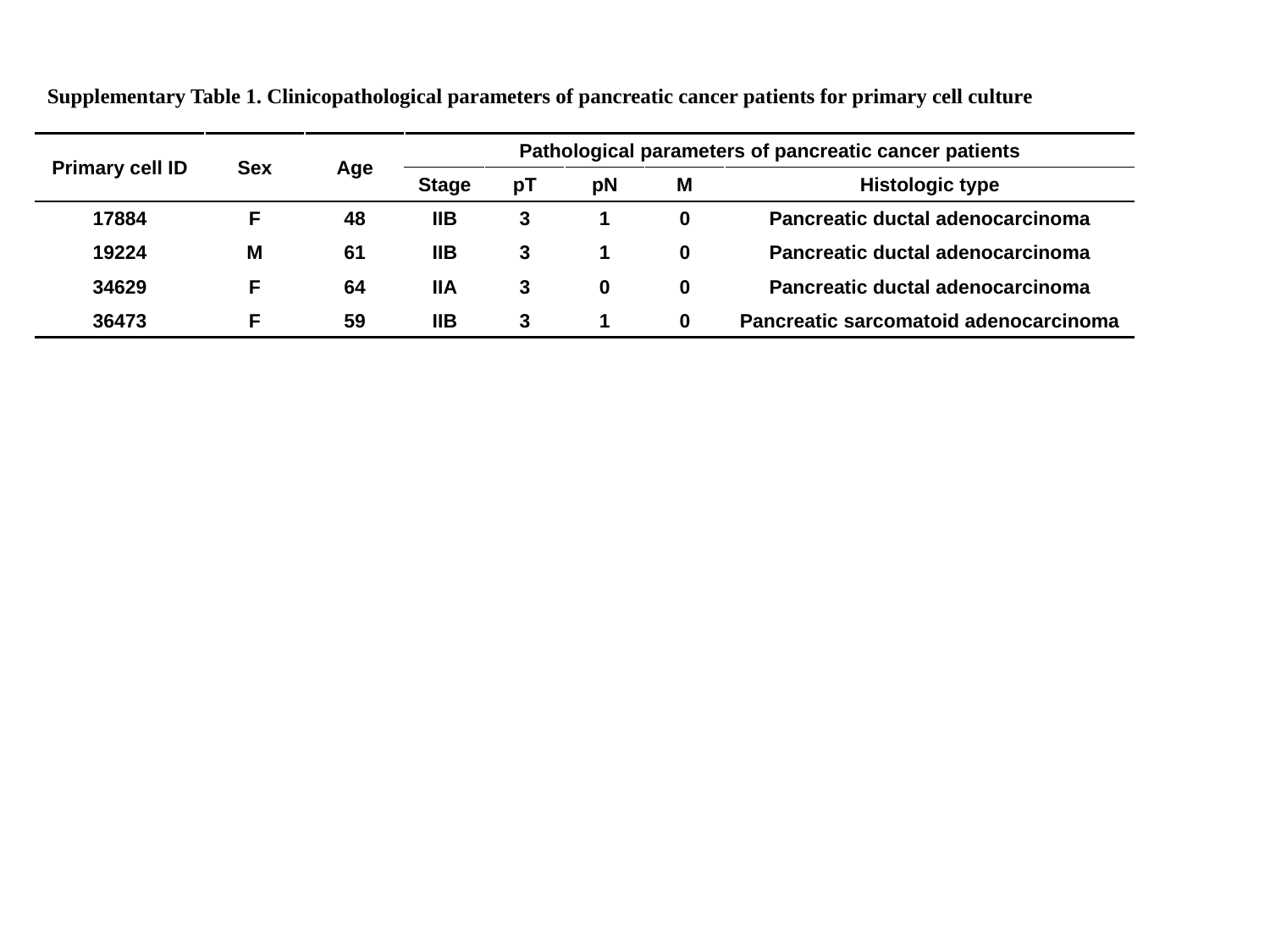

Supplementary Table 1. Clinicopathological parameters of pancreatic cancer patients for primary cell culture
| Primary cell ID | Sex | Age | Pathological parameters of pancreatic cancer patients | | | | |
| --- | --- | --- | --- | --- | --- | --- | --- |
| | | | Stage | pT | pN | M | Histologic type |
| 17884 | F | 48 | IIB | 3 | 1 | 0 | Pancreatic ductal adenocarcinoma |
| 19224 | M | 61 | IIB | 3 | 1 | 0 | Pancreatic ductal adenocarcinoma |
| 34629 | F | 64 | IIA | 3 | 0 | 0 | Pancreatic ductal adenocarcinoma |
| 36473 | F | 59 | IIB | 3 | 1 | 0 | Pancreatic sarcomatoid adenocarcinoma |

## Slide 13
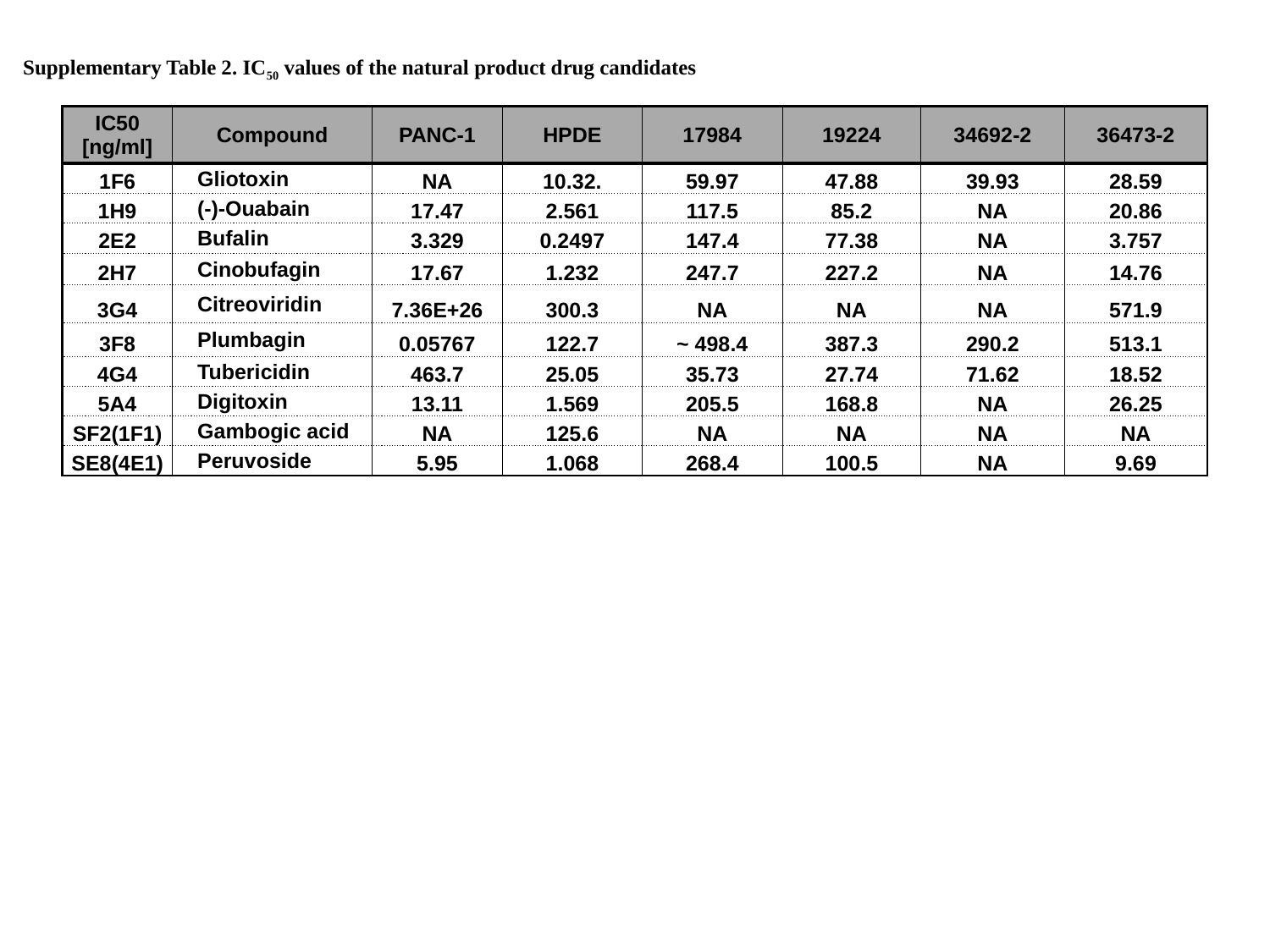

Supplementary Table 2. IC50 values of the natural product drug candidates
| IC50 [ng/ml] | Compound | PANC-1 | HPDE | 17984 | 19224 | 34692-2 | 36473-2 |
| --- | --- | --- | --- | --- | --- | --- | --- |
| 1F6 | Gliotoxin | NA | 10.32. | 59.97 | 47.88 | 39.93 | 28.59 |
| 1H9 | (-)-Ouabain | 17.47 | 2.561 | 117.5 | 85.2 | NA | 20.86 |
| 2E2 | Bufalin | 3.329 | 0.2497 | 147.4 | 77.38 | NA | 3.757 |
| 2H7 | Cinobufagin | 17.67 | 1.232 | 247.7 | 227.2 | NA | 14.76 |
| 3G4 | Citreoviridin | 7.36E+26 | 300.3 | NA | NA | NA | 571.9 |
| 3F8 | Plumbagin | 0.05767 | 122.7 | ~ 498.4 | 387.3 | 290.2 | 513.1 |
| 4G4 | Tubericidin | 463.7 | 25.05 | 35.73 | 27.74 | 71.62 | 18.52 |
| 5A4 | Digitoxin | 13.11 | 1.569 | 205.5 | 168.8 | NA | 26.25 |
| SF2(1F1) | Gambogic acid | NA | 125.6 | NA | NA | NA | NA |
| SE8(4E1) | Peruvoside | 5.95 | 1.068 | 268.4 | 100.5 | NA | 9.69 |
